# Critical reappraisal of short-acting bronchodilators for pediatric respiratory diseases

**DOI:** 10.1186/s13052-024-01675-0

**Published:** 2024-05-23

**Authors:** Amelia Licari, Sara Manti, Francesco Mastellone, Michele Miraglia Del Giudice, Gian Luigi Marseglia, Maria Angela Tosca, Asthma and Personalized Therapies Committee of the Italian Society of Pediatric Allergy & Immunology (SIAIP), Beatrice Andrenacci, Carlo Capristo, Renato Cutrera, Maria Elisa Di Cicco, Vincenzo Fierro, Maddalena Leone, Matteo Naso, Ilaria Pezone, Chiara Trincianti

**Affiliations:** 1 Department of Clinical, Surgical, Diagnostic and Pediatric Sciences, Pediatric Unit, University of Pavia, Pavia, Italy; 2 Pediatric Clinic, Fondazione IRCCS Policlinico San Matteo, Pavia, Italy; 3 Department of Human Pathology of Adult and Childhood Gaetano Barresi, Pediatric Unit, University of Messina, Messina, Italy; 4 Department of Life Sciences and Public Health, Post-Graduate School of Pediatrics, Policlinico Gemelli Universitary Foundation IRCCS, Catholic University of Sacre Hearth, Rome, Italy; 5 Department of Woman, Child and General and Specialized Surgery, University of Campania Luigi Vanvitelli, Naples, Italy; 6 Allergy Center, IRCCS Istituto Giannina Gaslini, Genoa, Italy

**Keywords:** Short-acting bronchodilators, Children, Bronchiolitis, Wheezing, Asthma

## Abstract

Short-acting bronchodilators are a class of medications commonly used to treat asthma, chronic obstructive pulmonary disease, and other respiratory conditions. The use of these medications has evolved over time as we have gained a better understanding of their effectiveness and safety in the pediatric population. This comprehensive review synthesizes the current understanding of short-acting β2-agonists and short-acting anticholinergics in children. It addresses indications, contraindications, safety considerations, and highlights areas where further research is needed to guide the most effective use of short-acting bronchodilators.

**Supplementary Information:**

The online version contains supplementary material available at 10.1186/s13052-024-01675-0.

## Introduction

Short-acting bronchodilators are a class of medications commonly used to treat acute and/or respiratory conditions such as asthma and chronic obstructive pulmonary disease. Their use has undergone continuous variations over the years due to an increase in knowledge regarding the effectiveness and safety of these drugs. Optimization in the use of these molecules, primarily used for bronchodilation, is essential for clinical practice. The objective is to maximize effectiveness while minimizing adverse effects.

In 2019, 12.9 thousand (95% UI uncertainty intervals (UIs) 10.6 to 15.7 thousand) children in the world died from asthma [1]. It has long been known that incorrect prescribing of short-acting β2-agonists (SABA), whether in excess or deficiency, results in increased asthma-related mortality [2]. The GINA document identifies using as-needed SABA more than twice a week as one indicator of poorly controlled asthma. Research suggests that dispensing more than three SABA canisters per year (corresponding to an average use of more than daily) is associated with an increased risk of emergency department visit or hospitalization regardless of asthma severity, and dispensing more than twelve canisters per year is associated with substantially increased risk of death [3–5]. The SABA use IN Asthma (SABINA) studies, a series of global research projects investigating the use of SABA in asthma, showed that to a considerable proportion of individuals with asthma in European countries are prescribed at least three SABA canisters per year [6]. Italy showed a lower SABA prescription rate than other European countries. Of the 22,201 patients enrolled, including all levels of asthma severity, a prevalence of 9% overuse of SABA was found [7]. However, this value might be underestimated as Italian regulations permit direct purchasing of SABA inhalers from pharmacies without a prescription [8]. Interestingly, 15% of participants acquired SABA without a prescription. Among those with a prescription, frequent SABA use (more than 2 canisters/year) was linked to a 30% higher risk of asthma exacerbations. To gain a more comprehensive understanding of SABA utilization, a subsequent analysis revealed an average annual use of 4 canisters among participants who reported purchasing SABA without a prescription [7].

The results of the SABINA study conducted in the pediatric population were consistent with previous studies in adults and adolescents and confirmed the association between high SABA use and increased asthma exacerbation risk in children; the study also highlighted the importance of monitoring SABA use in children, particularly those without asthma [9]. Overuse of SABA in children with asthma can also lead to several serious health risks, including decreased lung function, increased risk of side effects, and dependence on SABA.

Recently, the indications for short-acting bronchodilators use in the pediatric age have been changed in Italy, also because of numerous safety reports, and are intended to help prevent their overuse in children.

This review offers a comprehensive and critical analysis of recent research investigating the effectiveness and safety of SABA, with specific emphasis on salbutamol and ipratropium bromide, in the management of pediatric respiratory conditions. The primary aims of this review are to: evaluate the latest evidence on SABA effectiveness and safety in children, identifying both benefits and risks; outline optimal SABA use strategies tailored to specific pediatric respiratory diseases, considering dosage and delivery variations; identify critical knowledge gaps to guide future research focused on refining SABA use in children for maximum benefit and minimal risk.

A detailed description of the search strategy, including databases searched, search terms employed, inclusion and exclusion criteria, and data analysis methods is provided in Additional File 1.

## Therapeutic class overview of short-acting bronchodilators

### Short-acting beta2-agonists

SABA acts on β-adrenergic receptors (ARs), a type of postsynaptic receptor coupled to Gs proteins. ARs are classified into three types: β 1, β 2, and β 3, and are stimulated by noradrenalin and adrenaline [10]. β1-receptors have a high affinity for noradrenaline and adrenaline and can be found in the heart, brain, and adipose tissue. β2-Receptors show a lower affinity for noradrenaline and are involved in relaxing vascular and smooth muscles [11]. Β2-ARs constitute a major population of β-adrenergic receptors within the pulmonary system [10]. The relative selectivity of β2-agonists on β-ARs explains the systemic side effects caused by β2-agonists (i.e., tachycardia).

Β2-agonists trigger relaxation of airway smooth muscle (ASM) through a G protein-coupled signaling cascade. Upon binding β2-agonist ligand, the β2AR activates Gs protein, which stimulates adenylate cyclase to convert ATP to the key second messenger, cyclic adenosine monophosphate (cAMP). In particular, cAMP then activates two key downstream pathways: i) Protein Kinase A (PKA) pathway: PKA phosphorylates various regulatory proteins in ASM, including IP3, sarcolemmal Ca^2+^ channels, and proteins affecting myosin light chain phosphatase (MLCP) activity. MLCP dephosphorylation of the regulatory myosin light chain (rMLC) ultimately promotes smooth muscle relaxation; ii) Exchange Protein Activated by cAMP (Epac) Pathway: Epac acts as a GEF for Rap GTPases, leading to RhoA downregulation. This reduces rMLC phosphorylation and contributes to ASM relaxation independently of PKA [10].

Beyond cAMP, β2AR activation also triggers intracellular Ca^2+^ ([Ca^2+^]i) sequestration, reducing [Ca^2+^]i dynamics and sensitivity, which further promotes relaxation. Additionally, β2ARs enhance Ca^2+^-activated potassium channels (KCa^2+^), hyperpolarizing the cell membrane and facilitating bronchodilation [10, 12].

Clinically used β2-agonists share a core structure: a benzene ring linked to an amine head group by a two-carbon chain. The amine group can be unsubstituted or possess various substituents. Specificity within this class arises from variations in both the amine group and the benzene ring substitution patterns. These subtle structural modifications significantly impact the pharmacological profile by influencing the interaction with the β2-receptor and leading to differences in potency, duration of action, and selectivity [10]. The main SABA are listed below.

Salbutamol (called albuterol in the United States of America) is a selective β2-agonist. Traditionally, salbutamol was thought to have minimal to no effect on β1 receptors. However, recent research suggests there might be some β1 activity, even at higher doses [13]. Following inhalation, the initial bronchodilator effect becomes noticeable within 5–15 min. After the initial onset, the bronchodilator effect continues to improve, reaching its peak within 30–60 min. However, salbutamol weakly binds to the receptor and, consequentially, has a short duration of action (from 4 to 6 h).

Fenoterol is a resorcinol derivative considered relatively selective for β2ARs. Studies suggest that fenoterol may be 25 times more potent than salbutamol at the site of action. Like other β2-agonists, fenoterol exhibits a rapid onset of action (within 5–15 min) and a relatively short duration of action (3–4 h) [14].

Terbutaline is a synthetic sympathomimetic amine. Terbutaline shares the same mechanism of action as fenoterol, binding to β2ARs in airway smooth muscle and triggering the cAMP-PKA pathway to induce relaxation and bronchodilation. Additionally, terbutaline may have some inhibitory effects on the release of inflammatory mediators from mast cells, offering potential anti-inflammatory benefits.

It has a duration of action of 4 to 6 h [15].

Excessive or prolonged use of SABA can lead to tachyphylaxis, a phenomenon where the medication loses its effectiveness over time. This results in a gradual decrease in the bronchodilator effect of SABA, leading to less effective symptom relief. While the precise causes of SABA tachyphylaxis are still being elucidated, several potential mechanisms have emerged, such as the downregulation of β2-ARs, the desensitization of G protein-coupled signaling, and the refractory period of ASM cells [10, 14].

### Short-acting anticholinergic

ASM tone is mainly controlled by the parasympathetic nervous system via cholinergic mediators such as acetylcholine (ACh). ACh exerts its effect by primarily activating muscarinic M3 receptors (M3Rs) on ASM cells, airway epithelial goblet cells and submucosal glands, activating smooth muscle contraction and mucus secretion [16, 17]. In addition, airway exposure to ACh can trigger inflammation, potentially leading to the release of chemoattractants for eosinophils [17]. Antimuscarinic agents act via mAchRs inhibition. As a result, they induce airway bronchodilatation and reduce mucus production.

As well as their progenitor (atropine methonitrate, scopolamine), ipratropium bromide and oxitropium bromide are effective short-acting anticholinergic drugs used in treating asthma. Their duration of action is approximately 4 to 6 h, but compared to SABA, they have a slower onset of action [10, 16]. Ipratropium bromide is an active quaternary derivative of noratropine and is a nonselective antagonist of M1, M2, and M3 mAChRs. It has low lipid solubility and does not pass the blood–brain barrier; consequently, it does not show central nervous system side effects. Serum concentrations are extremely low after inhalation, and peak serum concentration is achieved 3 h after administration. Its metabolites have little anticholinergic activity. Unlike SABA, ipratropium bromide has a slower onset of action (15–30 min following inhalation). Peak bronchodilation is typically achieved within 30–60 min [10, 16].

Unlike their targeted action in the airways, muscarinic receptors are widely distributed throughout the body. This necessitates careful consideration, as their blockade by short-acting anticholinergics can lead to side effects in tissues beyond the airways. Common examples include dry mouth (xerostomia) due to inhibition of salivary gland secretions and urinary retention resulting from impaired bladder function [16, 17].

## Short-acting bronchodilators: indications in pediatric respiratory diseases

Table 1 provides an evidence-based framework for short-acting bronchodilator therapy in pediatric patients, aiding clinicians in making informed decisions for the treatment of wheezing, bronchiolitis, and asthma.

**Table 1:** Evidence-based approach to short-acting bronchodilator therapy in pediatric respiratory diseases

Disease	Key Age Groups	Short-Acting Bronchodilator Indications	Notes
Wheezing	Infants/Toddlers, Children	Acute Attack: Salbutamol (limited evidence under 1 year) Consider Ipratropium Bromide in combination for moderate-severe exacerbations	Uncertain efficacy of Ipratropium Bromide alone
Bronchiolitis	Infants < 12 months	Not recommended for routine use due to lack of benefit and potential harm	Salbutamol previously considered as a single trial, but now discouraged
Asthma (mild)	Adolescents, Children 6–11 years	No longer SABA alone Preferred: As-needed ICS-formoterol Alternative: ICS + SABA as needed	Focus on reducing exacerbations rather than symptom relief only
Asthma (moderate)	Adolescents, Children 6–11 years	SABA for acute exacerbations	Maintenance therapy with ICS + formoterol or ICS + LABA
Asthma (acute attack)	All ages	Salbutamol first-line; ipratropium bromide can be added for moderate-severe exacerbations	IV β2-agonists only for extreme cases with close monitoring

### Wheezing

Wheezing is an “umbrella” term, including heterogeneous clinical conditions with different respiratory phenotypes. It has been estimated that approximately 50% of children experience at least one wheezing episode before age 6 years [18].

In accordance with the temporal pattern of symptoms, children suffering from wheezing can be classified as patients with (a) transient early wheeze, which occurs before 3 years of age and resolves by the age of 6 years in the absence of lung function impairment; (b) late-onset wheeze, which develops after 3 years of age, persists in childhood, commonly featured by atopic predisposition, reduced lung function, and bronchial hyperresponsiveness; (c) persistent wheeze, which starts in early life before 3 years of age and is associated with atopic predisposition, increased serum immunoglobulin (Ig) E levels, allergen sensitization and decreased lung function by school age [19]. Based on triggers and symptoms, wheezing can be classified as “episodic viral wheezing” (EVW) or “multiple trigger wheezing” (MTW) [20]. EVW is mainly diagnosed in preschool age and is associated with clinical evidence of a viral respiratory tract infection, commonly sustained by Rhinovirus, Respiratory Syncytial Virus (RSV), Coronavirus, human Metapneumovirus, Parainfluenza virus, and Adenovirus [20]. In children having MTW, wheezing can also be induced by other triggers such as tobacco smoke and allergen exposure. Sometimes, crying, laughter, or exercise induce wheezing. However, it's important to note that not all children with MTW experience recurrent wheezing. The frequency and severity of wheezing episodes can vary considerably among individuals with MTW [21].

Although there is no univocal approach to treating wheezing, salbutamol and ipratropium bromide appear helpful in acute wheezing attacks. For children older than 2 years of age, inhaled salbutamol is the established first-line therapy for acute wheezing episodes. It is typically administered via a pressurized metered-dose inhaler (pMDI) with a spacer for mild to moderate wheezing, while nebulization with oxygen delivery is reserved for severe attacks [22–24]. The efficacy of inhaled salbutamol for wheezing episodes in children under 1 year of age remains uncertain. As the initial episode of viral wheezing in this age group is typically diagnosed as bronchiolitis, current guidelines do not recommend the use of salbutamol due to lack of evidence of effectiveness and concerns about harm [22–26]. A recent study explored the short-term efficacy of inhaled salbutamol, both with and without oral corticosteroids, for severe rhinovirus-induced wheezing in hospitalized children under 2. While salbutamol dosage alone did not impact hospitalization duration, high-dose salbutamol combined with prednisolone reduced wheezing relapse frequency and prolonged time to relapse compared to on-demand salbutamol with prednisolone. These effects were not observed in the placebo groups. However, the authors acknowledged inconsistencies within their findings, indicating the need for further studies to strengthen the evidence base [27].

In their systematic review, including 13 systematic reviews and 56 clinical trials and 5526 patients (aged 0–18 years), Pollock et al. [28] showed the efficacy and safety of SABA delivered by pMDI in treating wheezing. Regarding children aged 0–3 years, authors included only one clinical trial performed on 28 patients, in which SABA use was associated with a significant decrease in clinical severity score and respiratory rate and an increase in oxygen saturation. However, it did not show effects on hospital admissions [28].

Moreover, in children younger than 2 years of age, the European Medicines Agency (EMA), the Food and Drug Administration (FDA), the Medicines and Healthcare Products Regulatory Agency (MHRA) and the Italian Medicines Agency (AIFA) consider the use of nebulized salbutamol off-label, since its effectiveness and safety have not been established [29–32]. This indication could be attributed to several factors, such as the presentation of initial wheezing in young children as acute bronchiolitis (limiting bronchodilator response), as well as potential medication errors (dosing, administration) or accidental drug substitutions [32]. SABA administration in children under 2 years may be warranted under exceptional circumstances, including cases of severe respiratory distress where alternative treatments are ineffective or unsuitable, the use of specific protocols designed for this age group, and direct supervision by a qualified pediatric specialist.

The overall evidence surrounding the use of ipratropium bromide for wheezing treatment is complex and not entirely conclusive. Its effectiveness may depend on factors like the cause of wheezing, severity of symptoms, and patient age. While several studies support the use of ipratropium bromide in combination with salbutamol for moderate to severe exacerbations, others have shown minimal or no added benefit, particularly in milder cases [15, 33, 34]. The Italian Guidelines for managing acute asthma attacks in children [35] advocate for the combined use of ipratropium bromide and salbutamol for moderate-severe exacerbations, based upon the findings of the following studies. A 2012 Cochrane review on anticholinergic therapy in acute pediatric asthma analyzed four studies (*n* = 173 children) and indicated that the use of anticholinergics as a standalone therapy for acute pediatric asthma was associated with significantly higher treatment failure rates compared to β_2_-agonists alone or combined anticholinergic and β_2_-agonist therapy (OR 2.65; 95% CI 1.2–5.88) [36]. However, the relatively small number of studies and participants included in the review can limit the statistical power to detect smaller treatment effects and may affect the generalizability of the findings [36]. Another Cochrane published in 2013 examined the efficacy of adding inhaled anticholinergics to SABA for treating acute asthma in children [37]. The analysis of 15 studies (*n* = 2497 children), primarily involving both preschool-aged and school-aged children, demonstrated that the addition of anticholinergics significantly reduced hospital admission risk (RR = 0.73; 95% CI 0.63 to 0.85). Furthermore, this combination therapy improved lung function, clinical scores, oxygen saturation, and reduced the need for additional bronchodilators. Notably, fewer children receiving anticholinergics plus SABA experienced nausea and tremor compared to SABA alone [37]. Studies included participants aged 4 months to 18 years, with eight specifically focused on preschoolers and three on children under 18 months.

In 2020, researchers conducted a thorough analysis of Cochrane Reviews to consolidate current knowledge on the efficacy and safety of second-line treatments for children experiencing acute asthma attacks [38]. Of the 67 pediatric studies included in the 13 analyzed reviews, only 16 (24%) focused specifically on children under two years of age, highlighting a paucity of data for this age group. The analysis identified several key findings. First, the addition of inhaled anticholinergics to SABA therapy demonstrated a moderate-certainty reduction in hospitalization risk for children experiencing acute asthma exacerbations. Second, regarding side effects, the combination appeared to reduce the risk of nausea (high-certainty evidence) and tremor (moderate-certainty evidence) compared to SABA alone, with no significant difference observed for vomiting (low-certainty evidence). However, the authors acknowledged the limitations of drawing definitive treatment recommendations due to the scarcity of comparative studies directly evaluating different second-line options for this population [38].

### Bronchiolitis

Bronchiolitis is a lower respiratory tract viral infection affecting infants and young children younger than 12 months [26]. The most common causative agent is *Respiratory Syncytial Virus* (RSV), followed by *Rhinovirus* (RV), *Parainfluenza virus*, *Metapneumovirus* (MPV), *Influenza virus*, and *Adenovirus*, alone or in the form of co-infection [39].

Clinically, patients with bronchiolitis show, firstly, symptoms of a viral upper respiratory infection, such as rhinorrhea, that progress to the lower respiratory tract with cough; dyspnea; polypnea; increased respiratory effort manifested as nasal flaring, grunting, use of accessory muscles or intercostal and/or subcostal chest wall retractions; low oxygen (O_2_) saturation levels, apnea; skin color changes; feeding difficulties; lethargy; and, rarely, fever. Auscultatory findings include crackles and/or wheezing [26].

Bronchiolitis is featured by an extensive inflammatory process with recruitment and proliferation of polymorphonuclear cells (neutrophils and eosinophils) and lymphocytes, necrosis of airway epithelial cells and ciliary impairment, edema of the airways and mucus production, resulting in bronchiolar obstruction, air trapping, with different degrees of lobar collapse [39].

Due to the lack of a specific etiological treatment for acute bronchiolitis, therapy includes general supportive treatment to contain systemic and pulmonary symptoms. Superficial nasal aspiration, feeding and hydration, and oxygen supplementation are the mainstay for managing bronchiolitis [26]. Evidence on deep nasal aspiration, inhaled bronchodilators, hypertonic solution, and adrenaline, nebulized and systemic corticosteroids, antibiotics, and chest physiotherapy are controversial and inconclusive [40]. Specifically, the use of inhaler bronchodilators is widely discouraged. Because mucous obstruction and airway oedema rather than bronchospasm cause wheezing, salbutamol and ipratropium bromide should not administer to infants with a diagnosis of bronchiolitis, as they do not decrease the duration of symptoms, nor improve O2 saturation nor reduce the length of hospital stay. On the other hand, there is a potential risk of harm from their administration [25, 32, 41]. In October 2014, AIFA restricted the use of salbutamol drops (5 mg/ml) in the pediatric population, not supporting its use in children younger than 2 years of age due to the escalation in adverse effects resulting from wrong administration route or dosing or drug exchange [32]. Although several measures were previously adopted, including an explanatory posology correlation table and warnings on the risk of overdose due to administration error, severe adverse reactions have been reported in children younger than 2 years of age. The latter was attributed to incorrect administration routes, dosage errors, and drug exchange with the use of salbutamol (solution to nebulize). Adverse reactions included severe tremors and tachycardia and required hospitalization. The symptoms were resolved after discontinuation of treatment [32]. Therefore, a single therapeutic trial with inhaled salbutamol should not take into consideration in treating infants with acute bronchiolitis, contrary to what was previously supported [42]. Obviously, salbutamol remains one of the safest and most effective drugs currently available when administered appropriately and within prescribed.

### Asthma

In recent years, the Global Initiative for Asthma (GINA) has significantly revised its recommendations regarding the use of SABA as reliever medication for asthma. Prior to 2019, GINA guidelines suggested as-needed SABA as the primary treatment modality for individuals with mild, intermittent asthma. This treatment strategy aimed to alleviate symptoms during acute exacerbations but did not target the underlying chronic inflammatory processes inherent to asthma, even in its milder presentations. Accumulating evidence has demonstrated potential risks associated with SABA-only treatment, including an increased risk of severe exacerbations and a lack of control over chronic inflammation. Consequently, GINA has updated its recommendations, now strongly advising against the use of SABA as a standalone treatment for asthma in all patients, regardless of disease severity. Regular or frequent use of SABA may increase the risk of asthma-related death, urgent asthma-related health care, and severe exacerbations [23]. However, the observed relationship between SABA utilization and asthma-related mortality demands investigation into potential mechanisms. Elucidating whether excessive SABA use is a causal factor in poorer outcomes, or merely a surrogate marker of underlying disease severity, requires further research.

#### Mild asthma

The updated document now recommends that all adults and adolescents with mild asthma should receive either a combination of inhaled corticosteroids (ICS)-formoterol as symptom-driven therapy ("ICS-formoterol as needed”) or an as-needed combination of ICS-SABA (“as-needed ICS-SABA”) [23].

The as-needed low-dose combination of ICS-formoterol is the preferred treatment approach for Step 1 (Evidence B). Multiple large randomized controlled trials (RCTs) demonstrated that the combination therapy is more effective than SABA for reducing severe exacerbations and emergency department visits or hospitalizations [43–46]. In a 52-week, double-blind trial involving patients 12 years of age or older with mild asthma experiencing exacerbations, the as-needed budesonide-formoterol provided better symptom control than as-needed terbutaline, a SABA, and resulted in lower glucocorticoid exposure than budesonide maintenance therapy [44]. A post hoc pooled analysis of two clinical trials, SYGMA 1 and 2, assessed the efficacy and safety of as-needed budesonide-formoterol in adolescents with mild asthma [46]. This as-needed combination resulted effective as budesonide maintenance in preventing exacerbations and could be associated with better growth velocity in younger adolescents. The study also found that adolescents in the as-needed budesonide-formoterol group were more likely to adhere to their treatment regimen than those in the as-needed terbutaline group [46].

While these RCTs support the superiority of as-needed budesonide-formoterol over SABA alone in reducing severe exacerbations and healthcare utilization [43–46], it's important to acknowledge certain limitations inherent in these studies. Consideration should be given to the potential for sponsor bias, as many of these trials were funded by pharmaceutical companies with a vested interest in the investigated therapies. Relatively short follow-up periods in some studies may limit conclusions about long-term safety and efficacy of as-needed combination therapy. Further research with extended observational windows is needed to fully assess long-term outcomes. While the post-hoc analysis suggests potential benefits for growth velocity in younger adolescents using as-needed budesonide-formoterol [46], this finding warrants dedicated studies specifically designed to investigate this outcome, with a focus on potential dose-dependent effects on growth. Overall, despite these limitations, the existing body of evidence provides strong support for the use of as-needed budesonide-formoterol in mild asthma. However, ongoing and future research should address these constraints to provide a more comprehensive understanding of this treatment's long-term risks and benefits.

The alternative treatment option for Step 1 is low-dose ICS taken whenever SABA is taken (Evidence B) [23]. While the evidence for this approach in Step 1 is limited to small studies involving patients eligible for Step 2 treatment, GINA acknowledged the importance of minimizing severe exacerbations and the challenges of adhering to regular ICS therapy in patients with mild asthma symptoms [47–49]. Determining the most effective risk-minimization strategy for mild, persistent asthma, considering both the potential benefits of daily ICS therapy and the adherence barriers, warrants further research.

Among children between 6 and 11 years with asthma symptoms, treatment options include taking ICS whenever SABA is needed, supported by indirect evidence from Step 2 studies involving separate inhalers in children and adolescents. One study demonstrated significantly fewer exacerbations compared with SABA-only treatment [48], while another study exhibited similar outcomes to physician-adjusted therapy with a lower average ICS dose (Evidence B) [49]. Regular ICS plus as-needed SABA is also a potential treatment option for this age group (Evidence B). However, the likelihood of poor adherence in children with infrequent symptoms should be considered [23]. Thus, shared decision-making between clinicians, parents, and children is crucial for determining the optimal treatment approach in mild asthma, necessitating careful consideration of individual patient factors and preferences.

#### Moderate asthma

In moderate asthma, maintenance and reliever therapy with low-dose ICS-formoterol is the preferred option for adults and adolescents [23]. This approach proved to be effective in minimizing severe exacerbations and achieving comparable asthma control levels with relatively low ICS doses, compared to a fixed ICS-long acting β2-agonists (LABA) combination for maintenance therapy alongside higher ICS doses, both with as-needed SABA use. While an alternative approach involves maintenance ICS-LABA with as-needed SABA rescue medication, this strategy might be considered in specific contexts for moderate asthma. However, it is crucial to prioritize adherence to the daily ICS-LABA regimen, as poor adherence can significantly increase the risk of exacerbations. Before prescribing SABA-inclusive therapy, clinicians must carefully assess the patient's commitment to their controller medication [23].

For children with moderate asthma, as-needed SABA remains the cornerstone of reliever therapy. Maintenance therapy typically consists of daily low-dose ICS and LABA as the preferred option. Alternatively, a medium dose ICS or a very low dose of ICS-formoterol maintenance and reliever therapy can be considered [23].

#### Acute attack of asthma

Salbutamol is the first-line treatment for acute exacerbation [23]. For mild-to-moderate attack, administering inhaled salbutamol (2–4 puffs every 20 min during the first hour) via metered-dose inhaler (MDI) with a spacer device is recommended (Evidence A). After the first hour, the dosage of inhaled salbutamol can be flexibly adjusted between 4–10 puffs every 3–4 h or 6–10 puffs every 1–2 h, depending on the child's clinical presentation. Additional inhaled salbutamol may be unnecessary if clinical improvement is demonstrated by decreased or resolved wheezing, normalized oxygen saturation, and reduced respiratory distress, as well as peak expiratory flow (PEF) is sustained above 60–80% of the predicted or personal best value [23]. In the case of a patient with moderate-to-severe exacerbation requiring oxygen supplementation, 2.5 mg to 5 mg salbutamol diluted in 3 mL of sterile saline solution can be administered by an oxygen-driver nebulizer. Once improving, salbutamol should be administered by an MDI with a spacer device [35].

For moderate-to-severe asthma exacerbations in children not fully responding to salbutamol, nebulized ipratropium bromide may be administered. Dosage and frequency vary with age: younger than 4 years (125–250 mcg every 20 min for up to 1 h), or 4 years and older (250–500 mcg every 20 min for up to 1 h). The ipratropium dose should be tapered to 4 to 6 administrations before discontinuation. Upon discontinuing ipratropium bromide, the salbutamol should be gradually reduced to 1–2 hourly intervals based on clinical assessment [35].

A meta-analysis published in 2021 investigated the efficacy of adding ipratropium bromide to salbutamol for managing acute asthma in a pediatric population. The analysis included 55 studies with 6,396 children and adolescents experiencing acute asthma attacks. The results demonstrated that combination therapy with ipratropium bromide and salbutamol significantly reduced the risk of hospitalization compared to salbutamol alone (RR: 0.79; 95% CI: 0.66–0.95; *p*-value: 0.01). The I^2^ statistic of 40% suggested moderate heterogeneity amongst the studies [33]. The benefit of adding ipratropium bromide to salbutamol therapy was most pronounced in subgroups with greater initial disease severity. Subgroup analysis demonstrated a significant decrease in hospitalization risk for participants with severe (RR: 0.73; 95% CI: 0.60–0.88; *p* = 0.0009; I^2^ = 4%) and moderate-severe (RR: 0.69; 95% CI: 0.50–0.96; *p* = 0.03; I^2^ = 3%) asthma exacerbations, compared to salbutamol alone. However, the authors acknowledged limitations due to publication bias, methodological heterogeneity, and the variable quality (very low to high) of the included evidence. This underscores the necessity for future well-designed, double-blind randomized controlled trials (RCTs) with larger sample sizes to definitively evaluate the efficacy of this combination therapy in pediatric and adolescent asthma populations [33].

Finally, the update of the GINA document confirms the indication for the administration of short-acting SABA and ipratropium bromide in the treatment of moderate-severe acute asthmatic attacks, with evidence of efficacy on hospitalization in adults (high-quality evidence) and in children/adolescents (moderate-quality evidence) and on the improvement of respiratory function parameters, compared to the use of SABA alone, in adults and adolescents (high-quality evidence) [23].

Current guidelines do not support the routine use of intravenous (iv) administration of β_2_-agonists in children with severe asthma exacerbation [23]. Research on iv β_2_-agonists in children is relatively limited compared to inhaled therapies [50]. Existing studies often have small sample sizes, methodological differences, and vary in the severity of exacerbations they address. Conflicting results exist regarding whether iv β_2_-agonists offer significant advantages in symptom relief and clinical outcomes compared to optimizing therapy with inhaled β_2_-agonists. Children may respond differently to iv β_2_-agonists based on age, underlying comorbidities, and other individual factors. This variability makes it difficult to establish universal guidelines. Also, concerns persist about the potential for serious side effects associated with iv administration, such as cardiac arrhythmias and metabolic disturbances [50]. Weighing these risks against the potential benefits is crucial, especially in younger children. However, some authors suggest its use in children with severe exacerbation unresponsive to initial treatment; thus, the recommended dose is a single bolus of 15 μg/kg (dilution: 200 μg/mL for central iv line; 10–20 μg/mL for peripheral iv line) over 10 min, followed by continuous infusion of 0.2 μg/kg /min. Higher doses (1–2 μg/kg/min up to 5 μg/kg/min) can be administered in unresponsive children. Given the potential for severe side effects, iv β_2_-agonist use in children with severe asthma exacerbations mandates diligent monitoring and careful oversight by healthcare specialists experienced in pediatric critical care. The risks associated with iv β_2_-agonists necessitate administration within an ICU. Continuous ECG monitoring, along with twice-daily serum electrolyte and lactate level assessments, are required [35].

Together with bronchodilators, systemic corticosteroids are a mainstay in the management of moderate-to-severe asthma exacerbations in children and adolescents. These medications exert a rapid anti-inflammatory effect, leading to symptom relief and improved lung function. Consequently, systemic corticosteroids can help prevent hospitalization and shorten recovery time. However, due to potential side effects, they are typically administered for a short course (usually 3–5 days) during the acute phase of the exacerbation [51].

Finally, children experiencing severe asthma exacerbations require immediate access to emergency medical care. Rapid intervention with bronchodilators, oxygen therapy, and potentially advanced respiratory support can be lifesaving, ensuring optimal patient outcomes.

## Data safety of short-acting bronchodilator administration in the pediatric population

### Salbutamol

Salbutamol is included in the Essential List of Medicines provided by the World Health Organization (WHO) since it is considered “one of the safest and most effective drugs currently available” [52]; however, side effects following its administration are well-known in the literature.

ß2-adrenergic receptors are expressed not only in the lung membranes but also in skeletal muscle, vascular, and liver cells; thus, SABA administration can induce other effects than bronchodilatation [53]. Moreover, since many salbutamol dosage forms are available, the side effects are different and largely described in the literature.

A recent systematic review with meta-analysis, including 5.000 adults and adolescents > 12 years of age over an observational 25-year period, showed that severe adverse events (AEs) and deaths were not increased in patients treated with SABA when administered appropriately and within prescribed limits (5% and 0.22% incidence, respectively) [53, 54]. Inappropriate salbutamol administration exceeding prescribed limits represents the major driver of the high incidence of associated AEs [53]. Studies indicated a correlation between worsening asthma symptoms and increased SABA self-administration by patients, leading to a heightened risk of AEs. However, the authors reported some limitations in their study due to publication and reporting bias and methodological heterogeneity (i.e., dosage and duration inconsistencies, patient population variability, and outcome measure discrepancies) [54]. In general, it should be mentioned that judicious administration of SABA requires strict adherence to prescribed dosage and frequency instructions. Patients must avoid exceeding these limits and should seek immediate medical attention if symptoms worsen.

Globally, the inhaled form represents the safest route of administration [55]. In contrast, a continuous administration of SABA, regardless of the route of administration, is most associated with an increase in the risk for AEs [54]. It has been suggested that patients receiving regular SABA can develop desensitization and downregulation of ß2-receptors, with a loss of bronchodilator response and increased proinflammatory effects responsible for the higher risk for AEs [56].

Regarding the respiratory system, SABA administration can cause “thick neck,” chest heaviness, pulmonary edema, and paradoxical bronchoconstriction, especially in patients with historical asthma [57–60].

SABA administration has been shown to impact skeletal muscle strength and functional capacity [54]. This can lead to ergogenic effects, potentially enhancing performance. However, it's important to recognize that these ergogenic effects, while desirable for athletes seeking a competitive edge, can be detrimental in clinical contexts where patients are managing respiratory conditions. SABA use in such patients can cause muscle tremors and myopathy (muscle weakness), significantly compromising their function and well-being [54]. However, these effects have been attributed only to the systemic SABA administration. At the same time, inhaled salbutamol has minimal AEs at therapeutic doses, although the dose should not exceed 1600 mcg/day or 600 mcg over 8 h [61].

Regarding the cardiovascular side effects of bronchodilators, inhaled SABA may cause tachycardia and peripheral cardiac vasodilation-induced reflex and tachycardia, especially in patients with concomitant cardiovascular disorders, severe hypoxemia, and low serum potassium [62, 63]. However, compared to non-selective ß-agonists, SABA use showed a reduced incidence in cardiac AEs since it is selective for ß2-agonists [64]. The study found that the primary risk factor for arrhythmia (supraventricular tachycardia) was the cumulative number of SABA doses administered. This risk was particularly elevated in children with underlying heart conditions, histories of arrhythmias, and fevers [65, 66].

Hypokalemia and increased serum levels of insulin, glucose, pyruvate, free fatty acids, and lactate have been found in patients receiving high doses of salbutamol, regardless of the route of administration [67]. Salbutamol causes hypokalemia primarily through β2-stimulation of the Na + /K + -ATPase pump in skeletal muscle, which shifts potassium intracellularly. Hypokalemia can be significant enough to produce electrocardiogram (ECG) changes such as QT prolongation and U-waves. The risk of hypokalemia is higher when salbutamol is provided simultaneously with corticosteroids [68] and theophylline [69]. Thus, electrolyte monitoring is mandatory for patients on high-dose salbutamol or receiving medications that can affect electrolyte levels. Regarding the increase in lactate levels, the onset of salbutamol-induced lactic acidosis must be considered when high doses of salbutamol are administered [70]. β2-adrenergic stimulation increases cAMP-mediated gluconeogenesis and lipolysis with an increased in serum glucose and conversion to pyruvate and lactate.

While tremors are a common side effect of salbutamol, it's important to be aware of the potential for more severe, though rare, neurological side effects. At the central nervous system (CNS), salbutamol may cause seizures, hallucinations, and anxiousness due to its ability to cross the blood–brain barrier. In contrast, salbutamol-induced tremors arise from peripheral muscular imbalances rather than CNS stimulation [71, 72]. Educating patients and caregivers on appropriate dosing, potential side effects, and when to seek medical attention can significantly improve outcomes and mitigate these serious AEs. Tumorigenicity has not been related to salbutamol administration [73].

### Ipratropium bromide

Ipratropium bromide and other inhaled mAChR antagonists are generally well-tolerated due to their poor absorption after inhalation. The most common AEs of ipratropium bromide are dry mouth, throat irritation, and cough. These side effects are usually mild and resolve spontaneously. Other less common side effects include urinary retention, blurred vision, constipation, and headache. In rare cases, ipratropium bromide can cause more severe side effects, such as allergic reactions, worsening of asthma, and paradoxical bronchospasm. Of note, a history of hypersensitivity reactions or prior episodes of paradoxical bronchospasm increases the risk of a similar reaction when using ipratropium bromide. Accidental eye contact with these agents can lead to pupil dilation and blurred vision, posing a significant risk for individuals with glaucoma [10].

Several studies report safety information linked to the use of inhaled ipratropium bromide in the pediatric population with asthma. In a prospective observational study investigating the safety of ipratropium bromide in pediatric asthma exacerbations, Nomura et al. reported a single case of mild vomiting among the 77 children treated [74]. This isolated finding suggests that emesis may be a rare AE associated with ipratropium bromide use; however, further research is necessary to establish a definitive link. The observed AE, mild vomiting, was infrequent and categorized as the mildest grade on the Common Terminology Criteria for Adverse Events (CTCAE) scale, indicating its minimal impact on the patient's overall well-being [74]. The favorable safety profile observed in this study is consistent with findings from a Cochrane Systematic Review, which reinforces the established safety and efficacy of ipratropium bromide in pediatric asthma treatment [37]. However, it's crucial to acknowledge that systematic reviews can be influenced by factors such as publication bias and methodological heterogeneity (e.g., variations in treatment regimens and patient populations). These factors should be considered when interpreting and generalizing the review's conclusions.

A recent meta-analysis of RCTs examining the safety of combined ipratropium bromide and salbutamol in pediatric asthma treatment found nausea to be the only consistently reported AE. Other AEs, such as dry mouth, tremors, and vomiting, were not associated with the combination therapy [29]. While nausea is a potential side effect, it tends to be mild and infrequent, and the benefits of improved asthma control generally outweigh this risk.

While ipratropium bromide demonstrates generally favorable tolerability in both adults and children, it's important to acknowledge the potential for variations in AE profiles between these age groups. Careful monitoring remains essential to identify any potential discrepancies. Also, healthcare providers should proactively report AEs associated with ipratropium bromide use. This diligence enhances the understanding of the drug's safety profile and aids in detecting potential rare or unexpected side effects.

## Conclusions

The use of short-acting bronchodilators has evolved over time as we have gained a better understanding of their effectiveness and safety. SABA are effective for treating acute asthma exacerbations and wheezing attacks. However, they are not recommended as the sole maintenance therapy for asthma because they can lead to overuse and increased risk of severe exacerbations. Short-acting anticholinergics can be used in combination with SABA to treat moderate-to-severe asthma exacerbations. Both SABA and short-acting anticholinergics are generally well-tolerated and show a good safety profile when administered appropriately and within prescribed limits. Tremors, tachycardia, and dry mouth are the most common AEs. However, they are usually mild and tend to resolve spontaneously. Optimizing the use of these molecules for bronchodilation is critical in pediatric clinical practice.

### Supplementary Information

Supplementary Material 1.

## Data Availability

Not applicable.

## Abbreviations

Ach: Acetylcholine
AEs: Adverse events
AIFA: Italian Medicines Agency
Ars: Adrenergic receptors
ASM: Airway smooth muscle
cAMP: Cyclic adenosine monophosphosphate
CTCAE: Common Terminology Criteria for Adverse Events
CNS: Central nervous system
ECG: Electrocardiogram
EMA: European Medicines Agency
EVW: Episodic viral wheezing
FDA: Food and Drug Administration
GINA: Global Initiative for Asthma
ICS: Inhaled corticosteroids
LABA: Long-acting β2-agonists
mAChRs: Muscarinic acetylcholine receptors
MHRA: Medicines and Healthcare Products Regulatory Agency
MTW: Multiple trigger wheezing
PKA: Protein kinase A
pMDI: Pressurized metered-dose inhaler
RCTs: Randomized controlled trials
RSV: Respiratory syncytial virus
SABA: Short-acting β2-agonists
SABINA: SABA use IN Asthma
WHO: World Health Organization
