# Supplementary material for: Critical reappraisal of short-acting bronchodilators for pediatric respiratory diseases
Source: Ital J Pediatr. 2024 May 23;50:104. doi: 10.1186/s13052-024-01675-0 (PMC11112862; doi:10.1186/s13052-024-01675-0)
Supplement: Supplementary file 1 — Supplementary Material 1. [file 13052_2024_1675_MOESM1_ESM.docx]

Additional file 1

Study Details: Search Strategy, Inclusion/Exclusion Criteria, and Analysis Methods

| Search Methodology  Keywords: A comprehensive search will employ terms including "short-acting bronchodilators," "beta-agonists," "anticholinergics," "asthma," "bronchiolitis," "wheezing," "pediatrics," and "children."  Databases: Primary research databases will include PubMed, MEDLINE, Embase, Cochrane Library, and Google Scholar. |
| --- |
| Study Selection  Inclusion Criteria:   - Studies published in the English language - Research focused on the utilization of SABAs in children with respiratory disorders - Studies ranging from randomized controlled trials and observational studies to systematic reviews and meta-analyses   Exclusion Criteria:   - Individual case studies - Animal studies - Research solely focused on adult populations |
| Data Extraction and Synthesis  Extraction: Key data points extracted from each study will include:   - Study design and methodology - Patient demographics - Specific SABAs investigated - Study outcomes (efficacy, safety, adverse effects) - Key findings and conclusions   Synthesis: A narrative synthesis approach will integrate the findings across studies, highlighting:   - Effectiveness of different SABAs in various pediatric respiratory conditions - Safety profiles and potential adverse effects - Considerations for optimizing SABA use in pediatric settings - Areas warranting further investigation |
